# Symptomatic Histologically Proven Necrosis of Brain following Stereotactic Radiation and Ipilimumab in Six Lesions in Four Melanoma Patients

**DOI:** 10.1155/2014/417913

**Published:** 2014-07-01

**Authors:** Stephanie Du Four, Angela Hong, Matthew Chan, Michail Charakidis, Johnny Duerinck, Sofie Wilgenhof, Wei Wang, Linda Feng, Alex Michotte, Meena Okera, Brindha Shivalingam, Gerald Fogarty, Richard Kefford, Bart Neyns

**Affiliations:** ^1^Neurosurgery, UZ Brussel, Campus Jette, Laarbeeklaan 101, 1090 Brussels, Belgium; ^2^Medical Oncology, UZ Brussel, Campus Jette, Laarbeeklaan 101, 1090 Brussels, Belgium; ^3^Melanoma Institute Australia, Poche Centre, 40 Rocklands Road, Sydney, NSW 2060, Australia; ^4^Crown Princess Mary Cancer Centre Westmead, University of Sydney, Sydney, NSW 2060, Australia; ^5^Medical Oncology, Royal Darwin Hospital, 105 Rocklands Drive, Tiwi, Darwin, NT 0810, Australia; ^6^Radiation Oncology, University of Sydney, Edward Ford Building, Physics Road, Camperdown, Sydney, NSW 2006, Australia; ^7^Westmead Clinical School, University of Sydney, Cnr Hawkesbury Road & Darcy Road, Westmead, NSW 2145, Australia; ^8^Neuropathology, UZ Brussel, Campus Jette, Laarbeeklaan 101, 1090 Brussels, Belgium; ^9^Department of Neurosurgery, Mater Hospital, 25 Rocklands Road, Crows Nest, NSW 2060, Australia; ^10^Department of Radiation Oncology, Mater Hospital, 25 Rocklands Road, Crows Nest, NSW 2060, Australia

## Abstract

Four cases previously treated with ipilimumab with a total of six histologically confirmed symptomatic lesions of RNB without any sign of active tumour following stereotactic irradiation of MBM are reported. These lesions were all originally thought to be disease recurrence. In two cases, ipilimumab was given prior to SRT; in the other two ipilimumab was given after SRT. The average time from first ipilimumab to RNB was 15 months. The average time from SRT to RNB was 11 months. The average time from first diagnosis of MBM to last follow-up was 20 months at which time three patients were still alive, one with no evidence of disease. These cases represent approximately three percent of the total cases of melanoma and ten percent of those cases treated with ipilimumab irradiated in our respective centres collectively. We report this to highlight this new problem so that others may have a high index of suspicion, allowing, if clinically warranted, aggressive surgical salvage, possibly resulting in increased survival. Further studies prospectively collecting data to understand the denominator of this problem are needed to determine whether this problem is just the result of longer survival or whether there is some synergy between these two modalities that are increasingly being used together.

## 1. Introduction

Melanoma brain metastases (MBMs) are common and can impact quality of life and mortality, especially through a tendency to hemorrhage [1]. Historically, MBM confers a poor prognosis, with a median overall survival of less than 6 months [2]. Radiation is often employed and stereotactic radiosurgery (SRS) or stereotactic radiation therapy (SRT) is increasing following a positive randomized clinical trial (RCT) [3]. Radiation necrosis of the brain (RNB) is a well-known late toxicity of SRS. Given the dismal prognosis of patients with MBM, RNB has not previously been of major concern [4, 5].

Ipilimumab in melanoma is being increasingly used following RCTs demonstrating increased survival [6–8]. Controlling MBMs is therefore even more important. We recently published RNB in the scenario of three patients with MBM exposed to ipilimumab and SRS from a single institution [9] but only one had histologically confirmed RNB. This report is of four additional cases from multiple institutions that developed symptomatic RNB that was histologically confirmed.

## 2. Materials and Methods

### 2.1. Case 1

**Figure 1:**
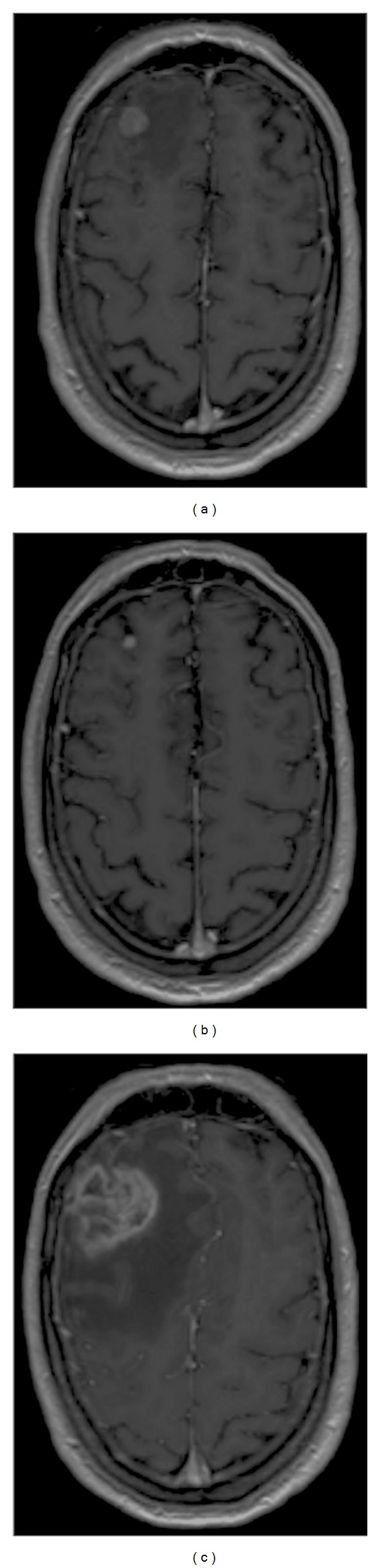
Gadolinium-enhanced T1-weighted MRI images obtained (a) at diagnosis, (b) 6 months after SRT, and (c) at occurrence of symptoms 9 months after SRT.

A 50-year-old male was treated with ipilimumab (3 mg/kg administered intravenously over 90 minutes every 3 weeks for a total of 4 administrations) for stage four disease 5 years after his primary diagnosis, resulting in disease stabilization. Two years later, reinduction of ipilimumab (3 mg/kg administered intravenously over 90 min every 3 weeks for a total of four administrations) was offered because of progression. A year later a new solitary right MBM (Figure 1(a)) was treated with SRS (1 fraction of 20 Gray (Gy)) with response over 6 months (Figure 1(b)). Treatment with the BRAF inhibitor dabrafenib was initiated at a dose of 150 mg twice daily for progression with extracranial disease that had a positive BRAF mutation.

**Figure 2:**
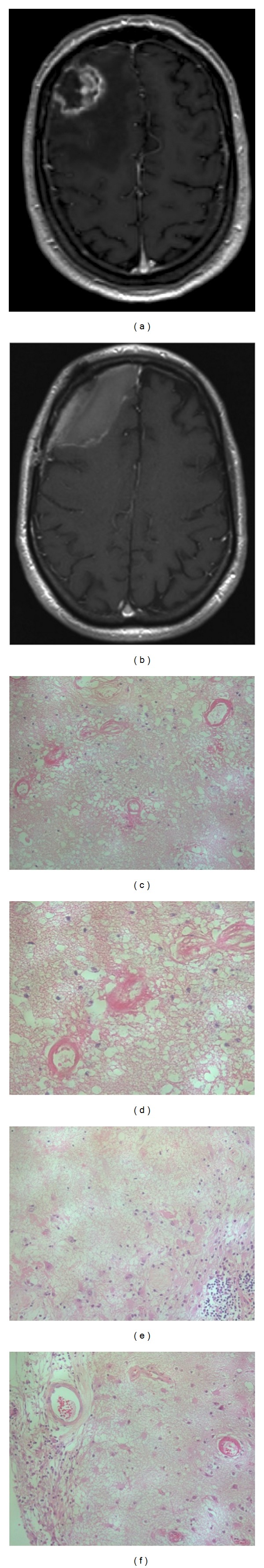
Gadolinium-enhanced T1-weighted MRI images obtained (a) 2 months after initiation of corticosteroids and (b) after surgical resections. Histopathological examination indicating (c) tissue necrosis and fibrinoid necrosis of small blood vessels (Hematoxylin and Eosine (H&E) staining, ×200); (d) H&E staining (×400); (e) radionecrosis surrounding gliosis and inflammatory cell reaction (H&E staining, ×200); and (f) focal infiltration of inflammatory cells and subpial gliosis (H&E, staining ×400).

Ten months later the brain lesion progressed (Figure 1(c)), accompanied by increasing headache and confusion without any other disease progression. RNB was suspected and treatment with methylprednisolone (32 mg, bid) and valproic acid (500 mg, bid) was initiated. Symptoms and perilesional oedema persisted at two months after the initiation of corticotherapy (Figure 2(a)). A neurosurgical resection confirmed the diagnosis of RNB (Figures 2(b) and 2(c)–2(f)). Eighteen months later the patient remains in complete remission on dabrafenib treatment.

### 2.2. Case 2

**Figure 3:**
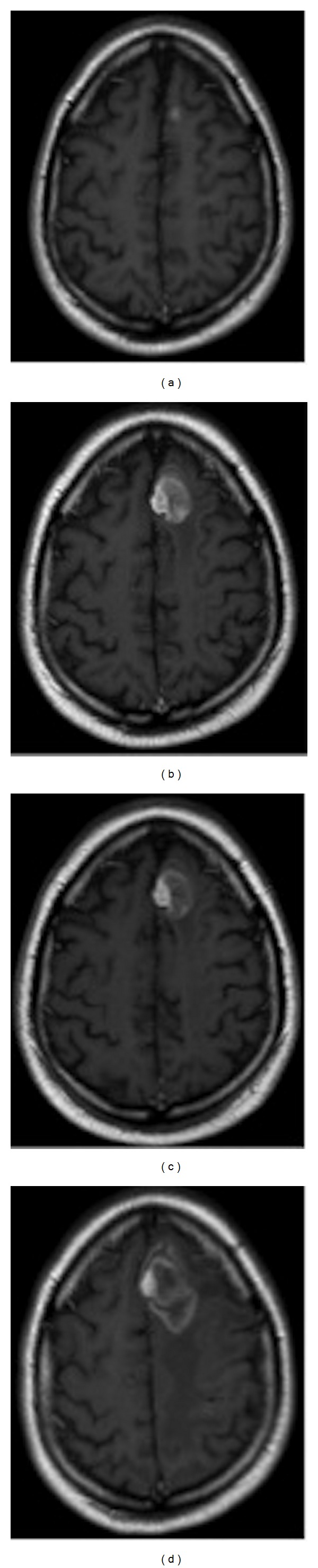
Gadolinium-enhanced T1-weighted MRI images obtained of the MBM of left frontal lobe (a) at diagnosis, (b) prior to SRT, (c) 3 months after SRT, and (d) prior to neurosurgery which revealed RNB only.

A 29-year-old man with stage four BRAF V600E positive disease had a solitary MBM in the left frontal lobe (Figure 3(a)) treated with surgery. Four months later, two new left frontal brain metastases had WBRT (30 Gy, 10 fractions) with a SRT boost (45 Gy in 10 fractions) (Figure 3(b) shows the first lesion at that time). Two months later ipilimumab was initiated (3 mg/kg administered intravenously over 90 minutes every 3 weeks for a total of four administrations). Four months later, a recurrent left frontal and new parietal lobe brain metastasis were treated with SRS (18 Gy in one fraction) (Figure 3(c)). Seven months later he developed headaches. Histology of the left frontal lobe metastases showed RNB (Figure 3(d)). At last follow-up six months later he then had progression.

### 2.3. Case 3

A 62-year-old man with unknown primary wild type BRAF metastatic melanoma had symptoms with three MBMs in the left temporal lobe, precentral gyrus, and frontal lobe treated with SRT (40 Gy in 8 fractions) [10, 11]. Three months later progressive extracranial disease was treated with ipilimumab (3 mg/kg administered intravenously over 90 minutes every 3 weeks for a total of four administrations). Six months later he presented with seizures and dysarthria, which were caused by an increase in the temporal lesion. Neurosurgical histology showed RNB. One month later he developed a right hemiparesis due to an increase in size of the lesion localized in the left precentral gyrus. Histology showed RNB. Six months later, sixteen months after the initial SRT, he re-presented with a worsening right hemiparesis due to a recurrence of the left precentral gyrus lesion. Histology revealed RNB. The patient died postoperatively from pulmonary embolus.

### 2.4. Case 4

**Figure 4:**
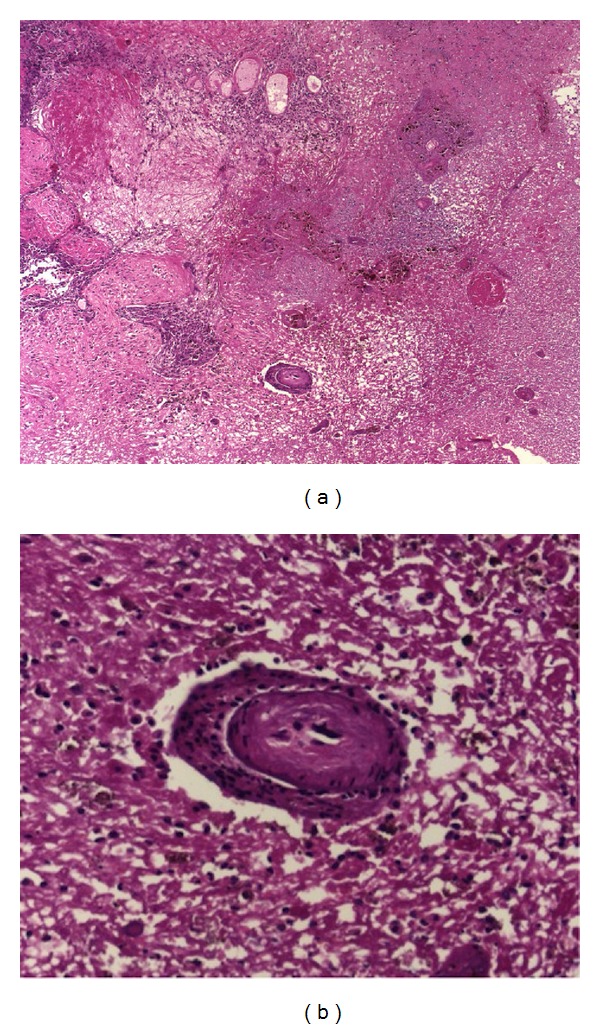
Histopathological examination indicating (a) an area of infarction (H&E staining, ×10) and (b) a transitional zone showing ischemia (H&E staining, ×20).

Two years after a BRAF wild type primary diagnosis, a 72-year-old man had a lung and three brain metastases in right frontoparietal, left parietal lobe, and left cerebellar hemisphere locations treated with WBRT (30 Gy in 10 fractions) with simultaneous integrated boost (45 Gy in 10 fractions) in a RCT. Three months later, ipilimumab was initiated (3 mg/kg or 10 mg/kg administered intravenously over 90 minutes every 3 weeks for a total of four administrations) to treat progressing extracranial disease. Fourteen months after RT he developed a headache and gait disturbance, with MRI showing enlargement of the right frontoparietal lesion. Neurosurgical resection showed RNB (Figure 4).

## 3. Results and Discussion

The results are summarised in Table 1. We report on four cases previously treated with ipilimumab with a total of of six histologically confirmed symptomatic lesions of RNB without any sign of active tumour following stereotactic irradiation of MBM. These lesions were all originally thought to be disease recurrence. The average time from first ipilimumab to RNB was 15 months. The average time from SRT to RNB was 11 months. In two cases ipilimumab was given prior to SRT, and in the other two ipilimumab was given after SRT. The average time from first diagnosis of MBM to last follow-up was 20 months at which time three patients were still alive, one with no evidence of disease. These cases represent approximately three percent of the total cases of melanoma and ten percent of those cases treated with ipilimumab irradiated in our respective centres collectively during the eight years of collection of these cases. We report this to highlight this new problem so that others may have a high index of suspicion, allowing aggressive surgical salvage if clinically warranted.

These four additional cases with RNB are of particular interest because RNB was histopathologically confirmed. In these cases, there was no marked infiltration of the resected tissue by inflammatory cells. This is different from the case reported by Hodi et al. [12] who documented marked infiltration by lymphocytes and macrophages in a resected MBM following SRS and ipilimumab treatment. Infiltration of melanoma metastases with immune cells (predominantly CD4^+^ and CD8^+^ T-cells) is a hallmark of the cellular response to ipilimumab treatment in responding patients. Such infiltration by immune cells can lead to an initial increase in the dimensions of the lesion, followed by regression. In our cases, high-dose corticotherapy preceding the resection of the lesion might have attenuated the inflammatory infiltrate. However, the absence of a symptomatic response to steroids and the fact that in the first case a nearly complete regression preceded progression of the lesion make it highly unlikely that an infiltration by immune cells was responsible for symptomatic progression.

The improved survival of patients responding to ipilimumab may place them at increased risk for developing late sequelae from treatment such as RNB. Peak incidence of RNB is around 12–15 months after radiotherapy. At 16–22 Gy, RNB can occur in up to 50% of treated lesions. For SRT the most important predictive variables include dose, tumour volume, and location of the lesion. The pathophysiological cascade that results in RNB consists of a series of inflammatory events, which leads to extracellular oedema, loss of neurons covered with myelin, and eventually hypoxia and necrosis [13–15].

Further studies prospectively collecting data to more exactly understand the denominator of this problem are needed to determine whether this problem is just the result of longer survival or whether there is some synergy between these two modalities that are increasingly being used together. The diagnosis and treatment of patients with new lesions in the brain, when successfully treated with SRS and immune therapies for melanoma, need to be approached with a high index of suspicion for RNB. Survival of these patients may be increased beyond that normally seen in MBMs by aggressive surgical salvage.

## 4. Conclusions

Four cases previously treated with ipilimumab with a total of six histologically confirmed symptomatic lesions of RNB without any sign of active tumour following stereotactic irradiation of MBM are reported. These lesions were all originally thought to be disease recurrence. The average time from first ipilimumab to RNB was 15 months. The average time from SRT to RNB was 11 months. The average time from first diagnosis of MBM to last follow-up was 20 months at which time three patients were still alive, one with no evidence of disease. These cases represent approximately three percent of the total cases of melanoma and ten percent of those cases treated with ipilimumab irradiated in our respective centres collectively during the eight years of collection of these cases. We report this to highlight this new problem so that others may have a high index of suspicion, allowing, if clinically warranted, aggressive surgical salvage, possibly resulting in increased survival.

## Figures and Tables

**Table 1:** Overview of the clinical history of the four patients.

Patient	1	2	3	4	Average months
MBM number	1	3	4	3	
MBM with RNB	1	1	3	1	
Dose of ipilimumab (mg/kg)	3	3	3	Blinded∗	
Reinduction of ipilimumab	Yes	No	No	No	
Type of RT	SRS	SRS + WBRT	SRT	SRT + WBRT	
Stereotactic radiation dose (total dose Gy/#)	20/1	18/1	40/8	45/10	
BRAF treatment before RNB	Yes	Yes	No	No	
Time from first ipi to RNB (months)	46	11	6, 7, 13	11	94/6 = 15
Time from SRT to RNB (months)	10	7	9, 10, 16	14	66/6 = 11
Further intracranial progression at last FU	No	Yes	No	No	
Status at last FU; months following first diagnosis of MBM	NED at 28	PD at 23	DONC at 16	PD at 14	81/4 = 20

## Abbreviations

MBM: Melanoma brain metastasis
SRS: Stereotactic radiosurgery
SRT: Stereotactic radiotherapy
WBRT: Whole brain radiotherapy
RNB: Radionecrosis of brain
FU: Follow-up
NED: No evidence of disease
PD: Progressive disease
DONC: Died of neurosurgical complications
RCT: Randomised controlled trial
Gy: Gray
#: Radiotherapy fraction; *either 3 or 10 mg/kg on RCT.
